# Prevalence and risk factors of feline leukaemia virus and feline immunodeficiency virus in peninsular Malaysia

**DOI:** 10.1186/1746-6148-8-33

**Published:** 2012-03-22

**Authors:** Faruku Bande, Siti Suri Arshad, Latiffah Hassan, Zunita Zakaria, Nurul Asyikin Sapian, Noor Alimah Rahman, Amer Alazawy

**Affiliations:** 1 Department of Veterinary Pathology and Microbiology, Faculty of Veterinary Medicine, Universiti Putra Malaysia, 43400 UPM Serdang Selangor, Malaysia; 2 Department of Veterinary Services, Ministry of Animal Health and Fisheries Development, PMB 2109 Usman Faruk Secretariat Sokoto, Sokoto State, Nigeria; 3 University Veterinary Hospital, Universiti Putra Malaysia 43400 UPM Serdang, Selangor, Malaysia

**Keywords:** Feline leukaemia virus, Feline immunodeficiency virus, Prevalence, Risk factors, Cats, Peninsular Malaysia

## Abstract

**Background:**

Feline leukaemia virus (FeLV) and feline immunodeficiency virus (FIV) are major causes of morbidity and mortality in domestic and wild felids. Despite the clinical importance of feline retroviruses and the growing interest in cats as pets, information about FeLV and FIV in Malaysia is presently insufficient to properly advise veterinarians and pet owners. A cross-sectional study was carried out from January 2010 to December 2010 to determine the prevalence and risk factors associated with FeLV and FIV among domestic cats in peninsular Malaysia. Plasma samples were harvested from the blood of 368 domestic cats and screened for evidence of FeLV p27 antigen and FIV antibodies, using an immunochromatographic kit. Additionally, data on cat demographics and health were collected using a structured questionnaire, and were evaluated as potential risk factors for FeLV or FIV status.

**Results:**

Of the 368 cats that were evaluated in this study, 12.2% (45/368; 95% CI = 8.88 - 15.58) were positive for FeLV p27 antigen, 31.3%, (115/368; 95% CI = 26.51 - 35.99) were seropositive to FIV antibodies, and 4.3% (16/368; 95% CI = 2.27 - 6.43) had evidence of both viruses. Factors found to significantly increase the risk for FeLV seropositivity include sex, age, behaviour, sickness, and living in a multi-cat household. Seropositive response to FIV was significantly associated with sex, neuter status, age, behaviour, and health status.

**Conclusions:**

The present study indicates that FeLV and FIV are common among domestic cats in peninsular Malaysia, and that factors related to cat demographics and health such as age, sex, behaviour, health status and type of household are important predictors for seropositive status to FeLV or FIV in peninsular Malaysia. High prevalence of FeLV or FIV observed in our study is of concern, in view of the immunosuppressive potentials of the two pathogens. Specific measures for control and prevention such as screening and routine vaccination are needed to ensure that FeLV and FIV are controlled in the cat population of peninsular Malaysia.

## Background

Feline leukaemia virus (FeLV) and feline immunodeficiency virus (FIV) are two clinically important retroviruses affecting domestic and non-domestic felids. FeLV is a gammaretrovirus, while FIV is a lentivirus [1,2]. Infection with FeLV or FIV is usually characterized by the development of lymphoma, anaemia, immunodeficiency, and secondary or opportunistic infections [3,4]. Transmission of FeLV occurs horizontally in nature through contact with the saliva and other secretions from infected cats. On the other hand, FIV is transmitted primarily through bite wounds [5,6]. Apart from the veterinary relevance of FeLV or FIV, the two pathogens provide useful models of human immunodeficiency virus (HIV-1) and human T-cell leukaemia virus (HTLV) [7,8].

FeLV and FIV are distributed widely among cats. Their prevalence varies geographically, and with associated risk factors such as age, sex, population density, and health status [6]. Although several studies have been carried out on important viruses of cats in Malaysia [9-11], little is known about seroprevalence of FeLV or FIV. Information on the prevalence of FeLV and FIV from different part of the world will be crucial in understanding the distributions and epidemiological parameters related to feline retroviruses worldwide. The aims of this study were to determine the seroprevalence of FeLV and FIV among domestic cats in peninsular Malaysia, and to evaluate the risk factors associated with positive serological responses.

## Results

### Prevalence

Out of the 368 cats tested in the present study, 12.2% (45/368; 95% CI = 8.88 - 15.58) were positive for FeLV p27 antigen, 31.3% cats (115/368; 95% CI = 26.51-35.99) were positive for FIV antibody, and 4.3% (16/368; 95% CI = 2.27-6.43) had evidence of both viruses. Prevalence of FeLV or FIV was greater in clinically sick (FeLV, 23.2%; FIV, 38.4%) compared to healthy cats (FeLV, 5.1%; FIV, 23.6%.), and in client-owned (13.1%) compared to shelter cats (10.3%). The seroprevalence of FeLV or FIV in each of the putative risk categories is presented in Tables 1 &2.

**Table 1:** Results of chi-square and univariate logistic regression analyses for risk of FeLV in peninsular Malaysia

Factors	Categories	Prevalence	*P *value	OR	95% CI
Sex	Female	12/163 (7.4%)	Ref	NA	NA

	Male	33/205 (16.1%)	0.011*	2.414	1.204 - 4.842

Neuter status	Intact female	6/116 (5.2%)	Ref	NA	NA

	Spayed female	6/46 (12.8%)	0.093	2.683	0.819-8.793

	Intact male	12/163 (17.4%)	0.469	1.356	0.592-3.105

	Castrated male	24/138 (13.4%)	Ref	1.131	NA

Age	Adult	24/251 (9.6%)	Ref	NA	NA

	Young	21/117 (17.9%)	0.022*	2.069	1.099-3.894

Breed	Domestic	36/305 (11.8%)	Ref	NA	NA

	Pedigree	9/63 (14.3%)	0.584	1.245	0.567 - 2.735

Household type	Single	3/68 (4.4%)	Ref	NA	NA

	Multi-cat	30/184 (16.3%)	0.013*	4.219	1.243-14.285

	Shelter	12/116 (10.3%)	0.156	2.500	0.6798 -9.174

Lifestyle	Indoor	13/134 (9.7%)	Ref	NA	NA

	Outdoor	20/184 (16.9%)	0.890	1.900	0.900-4.011

	Shelter	12/116 (10.3%)	0.866	1.074	0.470-2.456

Behaviour	Non-aggressive	27/281 (9.6%)	Ref	NA	NA

	Aggressive	18/87 (20.7%)	0.006*	2.500	1.300 - 4.808

Ownership	Shelter	12/116 (10.3%)	Ref	NA	NA

	Owned	33/252 (13.1%)	0.454	1.305	0.648 - 2.631

Sampling location	Other states	6/65 (9.2%)	Ref	NA	NA

	Selangor State	39/303 (12.9%)	0.416	1.452	0.588-3.590

FIV status	Negative	27/253 (10.7%)	Ref	NA	NA

	Positive	18/115 (15.7%)	0.176	1.553	0.817-2.952

Health status	Healthy	9/178 (5.1%)	Ref	NA	NA

	Sick	36/190 (18.9%)	< 0.001*	4.390	2.048-9.408

OR = Odds ratio; CI = confidence intervals; NA = not applicable; * = statistically significant difference; Ref = Reference category

**Table 2:** Results of chi-square and univariate logistic regression analyses for risk of FIV in peninsular Malaysia

Factors	Categories	Prevalence	*P *value	OR	95% CI
Sex	Female	41/163 (25.2%)	Ref	NA	NA
	Male	74/205 (36.1%)	0.024*	1.681	1.067 - 2.648
Neuter status	Intact female	35/116 (30.2%)	0.020*	2.951	1.149-7.576
	Spayed female	6/47 (12.8%)	Ref	NA	NA
	Intact male	53/138(38.4%)	0.323	1.366	0.735-2.538
	Castrated male	21/67 (31.3%)	Ref	NA	NA
Age	Young	28/117 (23.9%)	Referent	NA	NA
	Adult	87/251 (34.7%)	0.039*	1.686	1.025 - 2.777
Breed	Domestic	93/305 (30.5%)	Ref	NA	NA
	Pedigree	22/63 (34.9%)	0.490	1.223	0.690 - 2.168
Household type	Single	22/68 (32.4%)	Ref	NA	NA
	Multi-cat	55/184 (29.9%)	0.707	0.8912	0.490 - 1.621
	Shelter	38/116 (32.8%)	0.955	1.018	0.538 - 1.931
Lifestyle	Indoor	35/134 (26.1%)	Ref	NA	NA
	Outdoor	42/118 (35.6%)	0.103	1.563	0.912 - 2.680
	Shelter	38/116 (32.8%)	0.250	1.378	0.798 - 2.381
Behaviour	Non-aggressive	75/281 (26.7%)	Ref	NA	NA
	Aggressive	40/87 (46.0%)	0.001*	2.336	1.420-3.846
Ownership	Owned	77/252 (30.6%)	Ref	NA	NA
	Shelter	38/116 (32.8%)	0.672	1.107	0.691 - 1.774
Sampling location	Other states	15/65 (23.1%)	Ref	NA	NA
	Selangor state	100/303 (33.0%)	0.117	1.642	0.879 - 3.067
FeLV status	Negative	97/323 (30.0%)	Ref	NA	NA
	positive	18/45 (40.0%)	0.176	1.553	0.817 - 2.952
Health status	Healthy	42/178 (23.6%)	Ref	NA	NA
	Sick	73/190 (38.4%)	0.002*	2.020	1.284 - 3.178

OR = Odds ratio; CI = confidence intervals; NA = not applicable; * = statistically significant difference; Ref = Reference category

### Risk factors

In this study, several factors were significantly associated with FeLV or FIV seropositive status (Tables 1). In particular, risk of FeLV or FIV seropositive status was significantly higher in male cats, in aggressive cats, and in cats showing evidence of concurrent illness. Young cats were more likely to test positive for FeLV antigen while adult cats were more at risk for FIV seropositive status. Seropositivity to FeLV was more frequent in cats living in multi-cat households, compared to those living in shelters or single cat households. FIV antibodies were more prevalent among intact male and female cats, while positive response to FeLV testing was more frequent among intact males and neutered females. Factors such as breed, ownership, and sampling location, did not significantly influence FeLV or FIV seropositive status (p > 0.05).

## Discussion

### Prevalence

The prevalence and risk factors associated with FeLV or FIV were determined among domestic cats in peninsular Malaysia. Prevalence of FeLV or FIV in this study was greater than in the United Kingdom, North America, and Taiwan [12-14]. The observed differences might be attributed to variation among geographic regions, cat population densities, lifestyles and control policies and practices among different countries. Lack of routine vaccination against FeLV or FIV in Malaysia might be associated with high prevalence of feline retroviruses observed in our study. The impact of retrovirus infection has been drastically reduced in countries where vaccination, test-and-removal programs, and education campaigns are practiced [15,16].

### Risk factors

Seropositive response to FeLV or FIV was significantly higher in sick compared to healthy cats. Sick cats were 5 times more likely to test positive for FeLV p27 antigen, and 2 times more likely to be positive for FIV antibody. These observations are similar to studies carried out in the United Kingdom and Canada [12,17]. Since both FeLV and FIV are immunosuppressive in nature, it is likely that FeLV- or FIV-infected cats become predisposed to opportunistic or secondary infections [18].

The probability of seropositive response for FeLV or FIV was significantly higher among male cats. This is consistent with some epidemiological findings previously reported for retrovirus screening [13,19]. Danner et al. [20] reported that the sex of a cat significantly influences risk for FIV but not FeLV. On the other hand, Bandecchi et al. [21], observed no significant association between sex and seropositivity to FeLV or FIV. These variations might be related to difference in the type of cat populations being studied. For example, Danner et al. [20] exclusively sampled feral cats, while Bandecchi et al. [21] sampled owned cats. In contrast, our study included owned and shelter cats that probably had varying medical and behavioural characteristics [22].

In our study, cats with aggressive behaviour were 2 times more likely to test positive for FeLV or FIV, as compared with non-aggressive cats. A relationship between aggressive behaviours and seropositivity to FIV has been reported by several authors [19,23,24]. However, the high prevalence of FeLV observed in aggressive cats in our study contradicts the earlier notion that FeLV is primarily a disease of friendly or socialized cats [25]. Thus, there is the need to re-consider social behaviour in assessing FeLV transmission patterns [19,22]. It is possible that demographic factors that influence serological status to FeLV or FIV may change over time or with geography, climate and other factors.

FIV seropositive status was more frequent in intact male and female cats, compared to their neutered counterparts. On the other hand, positive response to FeLV testing was higher in intact males and neutered females. Reports of the relationship between sex and risk for feline retroviruses vary considerably. For example, in a study involving all parts of North America, FeLV or FIV seropositive outcomes were more frequent in intact females compared to spayed females, and in castrated males compared to intact males [13]. However, when only cats from the United State were considered, positive response to FeLV or FIV was observed more frequently among neutered male and female cats [26]. Greater seropositive status with respect to retrovirus infections in intact cats might be explained by their frequent involvement in territorial aggression and free-roaming behaviours that could increase risk for contact with infected cats. Although, in our study, seropositive test and odds of FeLV or FIV were greater among cats with outdoor access, this relationship was not significant statistically as reported recently in Canadian and German studies [17,19]. It is possible that lack of history on the original lifestyle of cats (indoor or outdoor) before their arrival at the shelter may affect our conclusion on the relationship between retrovirus seropositive response and lifestyle of cat population sampled. High prevalence of FeLV or FIV among free-roaming sexually intact cats might explain why the American Association of Feline Practitioners (AAFP) and The European Advisory Board for Cat Diseases (ABCD) recommended neutering as a means of reducing the frequency of feline retrovirus infections [27,28].

In our study, cats living in multi-cat households were most likely to be seropositive for FeLV, followed by cats living in shelters. FeLV seropositive status was least common among cats from single cat households. Conversely, seropositivity to FIV was not significantly influenced by type of household. This finding agrees with Fromont et al. [23], who also observed that prevalence of FeLV is more likely to be affected by population density compared to FIV. Overcrowding associated with multi-cat households often results in stress, poor hygiene, and increased direct contact among cats. FeLV transmission may likely be facilitated in these circumstances, since the virus is transmitted predominately by sharing of food and water containers [5,29].

The age of the tested cat was significantly associated with FeLV and FIV. Seropositive response to FeLV was 2.5 times higher in young cats, whereas seropositive response to FIV was 1.8 times higher in adult cats. This finding is in agreement with Levy et al. [13], and consisted with previous observations that increased susceptibility to FeLV was higher among young cats while susceptibility to FIV increases as cats grow older [30].

Although 4.3% of the sampled cat population was positive for both FeLV and FIV, we could not demonstrate any association between the two viruses statistically. There are conflicting opinions about the epidemiological relationship between FeLV and FIV. Some authors argued that FeLV and FIV occur independently [12,31], and others reported significant associations [19,32]. The debate notwithstanding, co-infection of cats with FeLV and FIV could lead to more negative health outcomes, compared to single infection with either virus [33].

It has been recommended that asymptomatic cats tested positive for FeLV p27 antigen should be re-tested within 6-12 weeks. This is because of low positive predictive value of single ELISA-based assays particularly in population with low seropositive rate [28,34]. No follow-up test was performed in the population we sampled, due to logistical problems and lack of willingness on the part of cat owners. In addition, cats in animal shelters usually remain for brief time periods as a result of adoptions and limited shelter space. Caution is therefore needed to avoid overgeneralizing the present findings.

## Conclusion

From the result of the present study, it was concluded that FeLV and FIV seropositive responses are high among cats in peninsular Malaysia and that seropositive status to FeLV and FIV is significantly influenced by several risk factors related to cat's demography and health. High prevalence of FeLV and FIV suggest the need for increased use of specific control measures such as screening and vaccination against feline retroviruses in peninsular Malaysia. As most of the identified risk factors are similar with those reported in other parts of the world, it is possible to adopt and implement current guidelines by the international associations for feline practices such as AAFP and ABCD to achieve better control and prevention strategies against feline retrovirus infections in peninsular Malaysia. Overall, this study has provided valuable insight on the occurrence of feline retroviruses in peninsular Malaysia, and has identified important risk factors.

## Methods

### Study area

The study was carried out in peninsular Malaysia which is located to the southern part of the Malay peninsula, extending from latitude l°20'N to 6°40'N and from longitude 99°35'E to 104°20'E. Peninsular Malaysia is the most densely populated part of Malaysia and covers an area of 124,450 Km^2^. The region can be divided into South, East, North, and Central regions. The climate is relatively humid (94-100%); with averagely high and uniform temperature (25-28°C) and rainfall (1,750 mm to 5,000 mm) throughout the year [35,36]. The people of this region have decades of history in keeping cats as their pets.

### Study design, participants and sampling

A cross sectional study was carried out to sample a total of 368 domestic cats from 12 veterinary hospitals or clinics (n = 252) and two animal protection shelters (n = 116) in peninsular Malaysia. Inclusion to this study was based on the willingness of veterinary hospitals and shelter management to participate in the study. In addition, consent was obtained from cat owners prior to the sampling. Sampling was carried out by convenience from January 2010 to December 2010. Blood samples of 1-3 mL were collected into EDTA tubes via jugular veinipuncture. All samples used in this study were collected by attending clinicians as part of routine practice.

### Testing protocol

Blood samples were centrifuged to collect plasma that was tested for the presence of FeLV p27 antigen and FIV antibodies, using an immunochromatographic kit (SensPERT FeLV Ag/FIV Ab kit). Testing protocols were as recommended by the manufacturer. The manufacturer reported test sensitivities for FeLV antigen and FIV antibody assays as 98.5% and 99.7%, respectively, while specificities for the assays were 97% and 99%, respectively (VetAll™, Korea).

### Risk factors

To evaluate risk factors associated with FeLV and/or FIV seropositivity, a structured questionnaire was completed for each sampled cat. Data recorded included sex, age (young [≤ 1 year] vs. adult [> 1 year]), breed (domestic [domestic short hair, domestic long hair, cross-breed] vs. pedigree), neuter status, household type (multi-cat household vs. single cat household or shelter), behaviour (aggressive vs. non-aggressive), health status (healthy vs. sick), lifestyle (indoor, outdoor, or shelter), ownership (owned vs. shelter), and sampling location (Selangor State vs. other States). Health status was evaluated from clinical records and general physical examination, while aggression was assessed based on cat demeanour as determined by the owners or shelter veterinarian.

### Data analysis

Information recorded on the questionnaires was arranged into Microsoft Excels 2007 spread sheet (Microsoft corporation Berkshire UK). Data was inspected and imported into an SPSS version 19.0 for statistical analysis (SPSS Inc. Chicago USA).

In the first step, descriptive statistics and frequency distributions were calculated and prevalence was determined as number of cats with positive serological test divided by the total number of cats evaluated. In addition, confidence intervals for each prevalence rates (FeLV, FIV and FeLV and FIV) were calculated at 95% level. Chi-square and univariate logistic regression analyses were used to determine association between the putative risk factors and seropositive response to FeLV or FIV. All statistical associations were considered significant at p < 0.05.

## Abbreviations

FeLV: Feline Leukaemia Virus; FIV: Feline Immunodeficiency Virus; CI: Confidence Interval; SPSS: Statistical Package for Social Sciences; UVH-UPM: University Veterinary Hospital Universiti Putra Malaysia; AAFP: American Association of Feline Practitioners: ABCD: Advisory Board for Cat Diseases.

## Competing interests

The authors declare that they have no competing interests.

## Authors' contributions

FB carried out the study, analysed the data, and drafted the manuscript. SSA conceived and coordinated the study, edited the manuscript, and approved the final submission. LH participated in the study design, data analysis, and proof-reading of the manuscript. ZZ participated in the study design and proof- reading of the manuscript. NAS, NAR, and AA participated in sample collection, study design, and proof- reading of the manuscript. All authors read and approved the final manuscript.
